# Crosstalk between CRISPR-Cas9 and the human transcriptome

**DOI:** 10.1038/s41467-022-28719-5

**Published:** 2022-03-02

**Authors:** Aaron A. Smargon, Assael A. Madrigal, Brian A. Yee, Kevin D. Dong, Jasmine R. Mueller, Gene W. Yeo

**Affiliations:** 1 Department of Cellular and Molecular Medicine, University of California San Diego, 9500 Gilman Drive, La Jolla, CA 92093 USA; 2 Stem Cell Program, University of California San Diego, Sanford Consortium for Regenerative Medicine, 2880 Torrey Pines Scenic Drive, La Jolla, CA 92037 USA; 3 Institute for Genomic Medicine, University of California San Diego, 9500 Gilman Drive, La Jolla, CA 92093 USA

**Keywords:** RNA, Targeted gene repair, CRISPR-Cas9 genome editing

## Abstract

CRISPR-Cas9 expression independent of its cognate synthetic guide RNA (gRNA) causes widespread genomic DNA damage in human cells. To investigate whether Cas9 can interact with endogenous human RNA transcripts independent of its guide, we perform eCLIP (enhanced CLIP) of Cas9 in human cells and find that Cas9 reproducibly interacts with hundreds of endogenous human RNA transcripts. This association can be partially explained by a model built on gRNA secondary structure and sequence. Critically, transcriptome-wide Cas9 binding sites do not appear to correlate with published genome-wide Cas9 DNA binding or cut-site loci under gRNA co-expression. However, even under gRNA co-expression low-affinity Cas9-human RNA interactions (which we term CRISPR crosstalk) do correlate with published elevated transcriptome-wide RNA editing. Our findings do not support the hypothesis that human RNAs can broadly guide Cas9 to bind and cleave human genomic DNA, but they illustrate a cellular and RNA impact likely inherent to CRISPR-Cas systems.

## Abstract (web summary)

The off-target effects of CRISPR-Cas9 are thought to be mediated by its cognate guide RNA. Here the authors show that Cas9 independently interacts with the human transcriptome, correlating with elevated RNA editing even under guide RNA co-expression.

## Introduction

CRISPR-Cas (clustered regularly interspaced short-palindromic repeats and CRISPR-associated proteins) systems have evolved in bacteria and archaea as adaptative immune systems defending against phage invaders^1^. Acquired foreign nucleic acids are stored in genomic memory as part of CRISPR arrays, which are then processed into CRISPR RNAs to which Cas proteins bind for downstream foreign nucleic acid recognition and destruction^2^.

The CRISPR-Cas9 system with its programmable synthetic guide RNA (gRNA)^3^ has developed into a powerful genome engineering and therapeutic tool^4^. It has been shown that Cas9 can associate with transcriptome-wide RNAs in bacteria, although such interactions were attributed to CRISPR RNA-mediated binding^5^. We hypothesized that Cas9 might also bind to endogenous eukaryotic RNA transcripts in a CRISPR RNA/gRNA-independent fashion, and that such interactions would be pervasive and potentially consequential in the far more complex transcriptome environment of human cells.

Here we show that Cas9 reproducibly binds to hundreds of human RNAs. These weak interactions in part observe a sequence and structure RNA motif modeled on the Cas9 gRNA. Although under gRNA co-expression such human RNA interactions do not correlate with Cas9 genomic DNA binding or cleavage, they do correlate with elevated RNA editing. Implications of this study for the use of CRISPR-Cas systems in human cells include off-target RNA editing or modification and bound transcript RNA/protein level changes.

## Results

### eCLIP identifies reproducible Cas9-human RNA targets

**Fig. 1:**
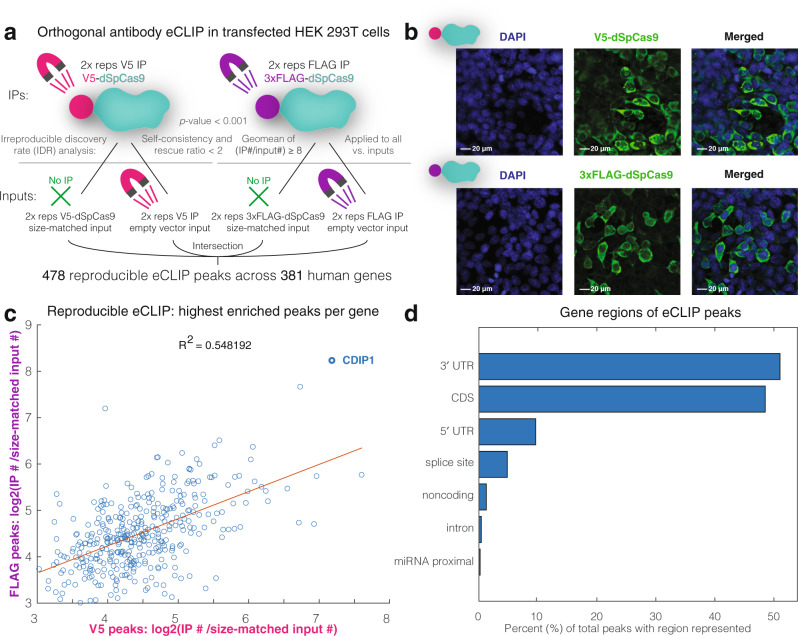
An orthogonal antibody eCLIP reveals transcriptome-wide Cas9-human RNA interactions. **a** Orthogonal V5/FLAG dSpCas9 eCLIP (enhanced eCLIP) experimental design. The experiment in transfected HEK 293T cells found 478 reproducible peaks across 381 human genes. All eCLIPs were performed in two bioreplicates per condition and were designed with two controls: size-matched inputs from dSpCas9 transfections; and antibody immunoprecipitations of empty vector transfections. **b** Immunofluorescent imaging of expressed V5/3xFLAG-dSpCas9 displays predominantly cytoplasmic cellular localization of dSpCas9 in HEK 293T cells. Experiments were performed independently in triplicate with similar results, with a representative image shown. **c** Highest enriched peak per gene log2(IP read count/size-matched input read count) enrichment score for V5 vs. FLAG eCLIPs. CDIP1(Cell Death Inducing p53 Target 1) is the top hit. **d** Gene regions of eCLIP peaks, with 3′ UTR the most represented.

To test our central hypothesis, we performed enhanced cross-linking and immunoprecipitation followed by sequencing (eCLIP) with anti-V5 and anti-FLAG antibodies in transfected human HEK 293T cells (Fig. 1a), as has similarly been conducted for human RNA-binding proteins (RBPs) with ineffective antibodies^6^. We selected cytoplasm-localized, catalytically dead dSpCas9 (Fig. 1b), given that it would be less likely to interfere with genomic DNA, to avoid confounding experimental effects. Performing two biologically replicate eCLIP experiments per condition and with two controls per antibody (no IP size-matched input; IP of transfected empty vector), we took the intersection of four Irreproducible Discovery Rate comparisons (self-consistency and rescue ratio < 2; geometric mean of IP read count:input read count ratio ≥ 8; *p*-value < 0.001) between experimental and control conditions. Out of this reproducible eCLIP dataset emerged 478 peaks across 381 human genes, with moderate correlation between V5 and FLAG eCLIP datasets (*R*^2^ = 0.548) and CDIP1 identified as the most enriched RNA substrate (Fig. 1c and Supplementary Data 1). Biological processes enriched in the dSpCas9-bound RNAs include genes in the categories cellular nitrogen compound biosynthesis, cytoplasmic translation, and peptide biosynthesis (Supplementary Fig. 1)^7^. Gene region analysis of peaks revealed that most dSpCas9-human RNA interaction sites occur within the 3′ UTR of coding mRNA, and just under 50% occur within the CDS (Fig. 1d), frequencies which may depend on their relative lengths and/or secondary structures represented in the RNA-sequencing dataset (Supplementary Fig. 2 and Supplementary Data 2).

### Cas9-bound human RNAs associated with p53 pathway

It has been reported in multiple publications that Cas9 expression in human cells induces a stress/p53 response associated with DNA damage^8–10^. Whether Cas9 may through RNA interactions activate the stress response pathway to induce apoptotic DNA damage remains an open question. To resolve this question, an examination of the RNAs encoding the 381 human genes with which Cas9 interacts uncovered 66 (17.3% of the eCLIP targets) to be associated with the stress response pathway, with CDIP1, ATF3, and CDKN1A (p21) among the top four enriched genes in our eCLIP experiment (Supplementary Fig. 3a). We analyzed data from a study profiling Cas9-mediated gene expression in a total of 165 human cancer cell lines with and without integrated Cas9^10^. To eliminate significant gene expression changes potentially due to data artifacts, we filtered out genes with less than 1 log2 RPKM L1000 expression in any of the 165 control (empty vector) cell lines^11^. Of the 55 stress response pathway genes that passed this filter, ATF3 (Activating Transcription Factor 3) emerged as the most Cas9-dependent upregulated gene (Supplementary Fig. 3b).

We confirmed this finding with quantitative reverse transcription PCR (RT-qPCR) in transfected HEK 293T cells, comparing the expression effects of dSpCas9 with and without U6 promoter-driven gRNAs, SpCas9-NLS (catalytically active with a nuclear localization signal), and the fluorescent protein UnaG^12^ (as a negative control against potential confounding protein agnostic translational stress effects) against an empty vector-negative control (Supplementary Figs. 3c and 4). Like ACTB and non-eCLIP substrate p53, Cas9-bound RNA targets CDIP1 and CDKN1A showed little or inconclusive mRNA expression changes across the different conditions. ATF3, which contains a Cas9 eCLIP peak in its 3′ UTR (Supplementary Fig. 3d), was consistently upregulated upon expression of each Cas9 condition, but not upon expression of the empty vector or fluorescent protein controls (one-way ANOVA pairwise *p*-values < 0.05). A stress pathway master regulator transcription factor, ATF3 is known to share DNA-binding sites with p53, with which it interacts cooperatively^13^. Moreover, ATF3 overexpression has been implicated in the acceleration of apoptosis in human HepG2 cells^14^. Western blot analysis revealed a moderate increase in ATF3 protein level upon cytoplasm-localized dSpCas9 expression without gRNA co-expression over both empty vector and fluorescent protein conditions (Supplementary Fig. 5). Therefore, it is conceivable that ATF3 upregulation through Cas9-ATF3 3′ UTR binding-mediated mRNA stabilization might in certain cellular contexts contribute to apoptosis and associated DNA damage in human cells.

### Biochemical mechanism of Cas9-human RNA interactions

**Fig. 2:**
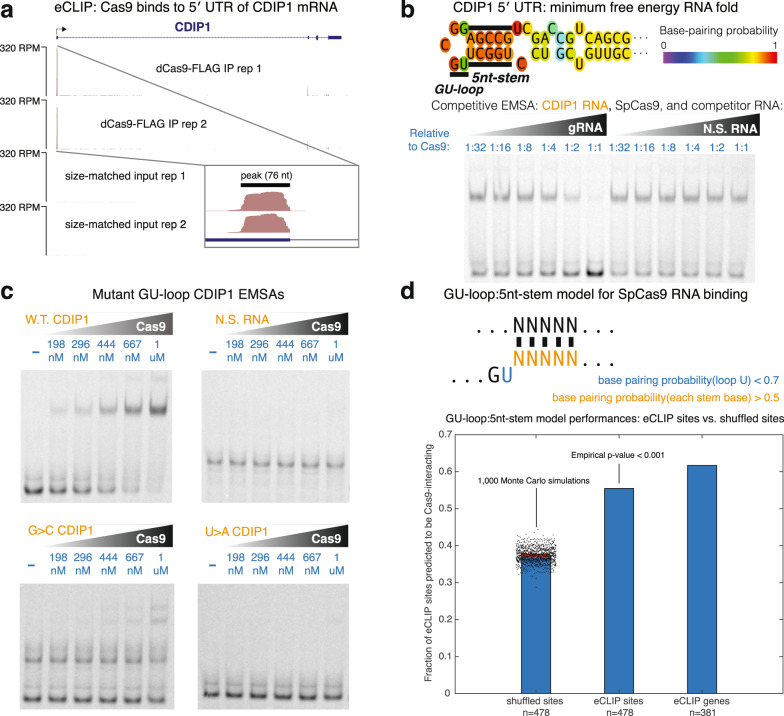
The CRISPR RNA recognition domain of Cas9 binds to human RNA. **a** dSpCas9 binds to the 5′ UTR of CDIP1 mRNA. **b** A minimum free-energy RNA fold (Vienna RNAfold) of the CDIP1 5′ UTR reveals a GU-loop:5nt-stem sequence-structure motif, identical to the SpCas9 gRNA. gRNA outcompetes 20 nM of 5′-fluorescently labeled CDIP1 RNA, whereas non-specific (N.S.) RNA does not, in a competitive EMSA (electrophoretic mobility shift assay). Experiments were performed independently in triplicate with similar results, with a representative gel shown. **c** SpCas9 protein binds to 20 nM of 5′-fluorescently labeled CDIP1 mRNA with an apparent dissociation constant (*K*_D_) in the high nanomolar range. Mutations to either the G or U of the GU-loop significantly reduce this binding affinity. Experiments were performed independently in triplicate with similar results, with a representative gel shown. **d** A GU-loop:5nt-stem model that searches an RNA sequence for a GU-loop motif (with the base-pairing probability of the U < 0.7) upstream of a 5nt-stem (five bases each with base-pairing probability > 0.5) trends with eCLIP peaks and genes predicted to interact with dSpCas9. The prediction of 1000 Monte Carlo simulations based on shuffled eCLIP peak sequences is represented by scatter, where the mean is bold horizontal red line, and 95% confidence interval is lighter red box. Empirical *p*-value < 0.001 (Monte Carlo simulation). (Invisible plotted confidence interval is within plotted mean).

We next sought to elucidate the biochemical mechanism of Cas9 binding to human RNA. Our top eCLIP hit, CDIP1, demonstrated binding by dSpCas9 to the 5′ UTR of its mRNA (Fig. 2a). A Vienna RNAfold minimum free-energy structure of this binding site indicates the presence of a GU-loop upstream of a 5-nucleotide RNA stem (Fig. 2b)^15^, identical to that of the gRNA to which the CRISPR RNA recognition domain of SpCas9 binds^16^. To confirm that this domain binds to the 5′ UTR of CDIP1 mRNA, we performed a competitive electrophoretic mobility shift assay of fluorescently labeled CDIP1 RNA with unlabeled gRNA and non-specific RNA, finding that gRNA—and not non-specific RNA—outcompetes CDIP1 RNA (Fig. 2b). Further validating this hypothesis, point mutations to either the G or U of the GU-loop effectively abolished an apparent high nanomolar (>100 nM) dissociation constant (*K*_D_) of SpCas9 for CDIP1 RNA (Fig. 2c), in contrast with a mid-picomolar *K*_D_ of SpCas9 for its gRNA^17^. Further mutations intended to disrupt the 5nt-stem RNA secondary structure likewise abolished the binding affinity. Surprisingly, truncating the loop while preserving its GU motif enhanced binding affinity, representative of the complex nature of protein-RNA interactions (Supplementary Fig. 6).

A computational model based on the GU-loop:5nt-stem (base-pairing probability of loop *U* < 0.7; base-pairing probability of each of the five stem bases > 0.5 in Vienna RNAplfold) showed increased performance in predicting Cas9-interacting eCLIP sites over Monte Carlo simulations (empirical *p*-value < 0.001) of the same nucleotide sequences randomly shuffled (Fig. 2d)^15^, with performance improving when collapsing peaks that share an identical gene. While somewhat encouraging, RNA secondary structures are notoriously difficult to predict in silico. In addition, Cas9 has been reported to bind to other regions of its gRNA with rival affinity^17^. Nevertheless, this model can partially account for gRNA-independent human transcriptome-wide Cas9 interactions. In further support, of the ten eCLIP binding sites that overlap in vivo click selective 2-hydroxyl acylation and profiling experiment structure probing data in HEK 293T cells, six contain a GU-loop:5nt-stem, and an additional structure contains a GU-loop:4nt-stem (Supplementary Fig. 7)^18–20^.

### Cellular impact of Cas9-human RNA interactions

Given that Cas9 reproducibly binds to hundreds of human RNA transcripts, we next asked whether these RNAs can guide Cas9 to induce DNA damage in human cells. To evaluate this possibility, we surveyed genome-wide Cas9-mediated DNA cleavage and catalytically dead Cas9 DNA-binding (by CHIP-seq) datasets in HEK 293T cells^21,22^. An examination of the frequency of cleavage events (unique dsODN tag inserts) in non-eCLIP genes vs. eCLIP targets showed no statistically significant differences when comparing a no-Cas9/no-gRNA-negative control to four Cas9 conditions with different gRNAs co-expressed (Supplementary Fig. 8). Likewise, in each of two replicates of three different gRNA conditions, no statistically significant elevated single-base maximum CHIP-Seq coverage was found in Cas9-bound RNA target genes vs. non-targets for genes (with an expression cutoff of TPM (transcripts per million) > 1; Supplementary Fig. 9a). Interestingly, maximum CHIP-Seq coverage (i.e., DNA-binding frequency) does correlate moderately and reproducibly with gene expression level for non-eCLIP genes (*R*^2^ values ranging from ~0.14 to ~0.26 across all replicates). Thus, regions of open chromatin may play the dominant role in Cas9 DNA-binding site preference (Supplementary Fig. 9b). Given these findings, while it is known that Cas9 expression in the absence of gRNA can induce genomic DNA damage in human cells, such damage may be predominantly driven by genomic DNA surveillance independent of an RNA guide intermediary—a phenomenon that has been demonstrated to be biochemically feasible^23^.

**Fig. 3:**
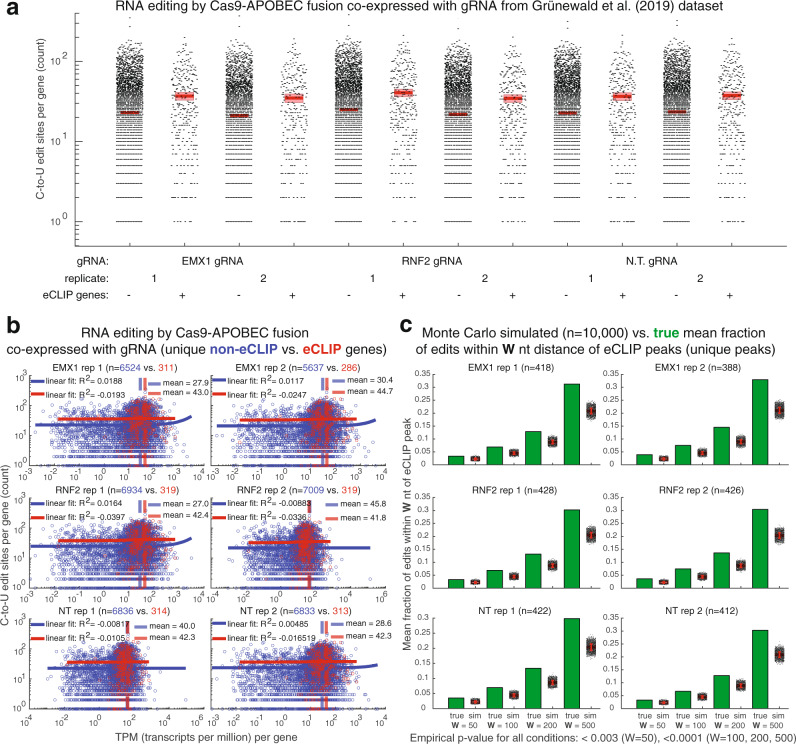
Cas9-interacting human RNA transcripts reproducibly correlate with elevated transcriptome-wide RNA-editing activities at their target genes even under gRNA co-expression. **a** Box plots of C-to-U edit site counts per gene, for non-eCLIP vs. eCLIP genes. Data for each experimental condition (two replicates of three different gRNAs co-expressed with Cas9-APOBEC fusion) are plotted only of genes with at least one edit site according to Grünewald et al.^25^ For each dataset mean is bold horizontal red line and 95% confidence interval is lighter red box. (Invisible plotted confidence intervals are within plotted means.) **b** Scatter plot based on **a** TPM (transcripts per million) per gene vs. C-to-U edit site counts per gene, for non-eCLIP (blue) vs. eCLIP (red) genes. Linear fits with *R*^2^ values of each dataset by gene type are plotted in solid lines, whereas TPM means of each dataset by gene type are plotted in dashed lines. **c** Plots of the predictions of Monte Carlo simulated (10,000 simulations) vs. true mean fraction of edits within W (50, 100, 200, 500) nt distance of each eCLIP peak represented in **a** whose center is on a spliced RNA transcript to each C-to-U edit site on that transcript. Empirical *p*-value <0.003 for *W* = 50 for all conditions; <0.0001 for *W* = 100, 200, 500 for all conditions (Monte Carlo simulation). For Monte Carlo simulations, simulated eCLIP peaks were placed along the spliced RNA transcript according to a uniform random distribution. True mean fractions of edits are represented by green bars, while the Monte Carlo simulations are represented by scatter, where the mean is a bold horizontal red line superimposed with red standard deviation error bars.

Cas9 and other CRISPR-Cas systems are widely employed not only as a DNA-editing tools, but also as RNA-editing tools^24^. For this reason, we analyzed a publicly available Cas9 RNA-editing HEK 293T dataset^25^. In each of two replicates of three different gRNA conditions, a nickase Cas9-APOBEC fusion produced statistically significant (outside 95% confidence intervals) more edit sites in eCLIP target gene transcripts vs. non-eCLIP gene transcripts (Fig. 3a). This effect cannot be explained by differences in RNA expression level, which are uncorrelated with RNA editing rates across both non-eCLIP and eCLIP genes (|*R*^2^ | values < 0.04 across all replicates) (Fig. 3b). If nickase Cas9-APOBEC fusion co-expressed with gRNA binds to and edits human RNA transcripts with which it also interacts in the absence of gRNA, we would expect RNA edit sites to cluster around eCLIP peak sites. In support of this, the mean fractions of C-to-U edits within sequence windows of 50, 100, 200, and 500 nt proximal to eCLIP peaks across replicates are significantly higher, relative to Monte Carlo simulated peaks across the represented transcripts (empirical *p*-value < 0.003 for *W* = 50; <0.0001 for *W* = 100, 200, 500) (Fig. 3c). This observation comports with our finding that APOBEC fusions to some RBPs can edit distances farther in linear space due to the dynamic and compact nature of mRNA conformations^26^. In the present study, differential RNA-editing profiles of eCLIP vs. non-eCLIP genes are especially notable, given the far higher affinity of Cas9 for its gRNA over even the most enriched eCLIP peak gene.

## Discussion

While the scope of this study concerns the established CRISPR-Cas9 system, Cas protein-human RNA interaction-mediated cellular effects, which we term CRISPR crosstalk, may have far-reaching implications for the CRISPR field (Supplementary Fig. 10). Despite the expression of synthetic guide RNAs in CRISPR-based transcriptomic engineering applications, we anticipate potential concerns with off-target binding and consequential editing/modifying by RNA-targeting CRISPR systems (e.g., Cas13) fused to RNA-modifying effector proteins, particularly because these CRISPR systems typically possess shorter and less complex Cas protein-interacting synthetic guide RNA structures than that of Cas9^27^.

It is unclear if CRISPR crosstalk represents a phenomenon with substantial impact on cellular fitness. We predict that CRISPR crosstalk for some CRISPR-Cas systems may have profound effects on bound transcript RNA and/or protein. Follow-up studies may show more direct links, although demonstrating clear causal effects from pleiotropic subtle RNA interactions comes with inherent challenges. Nonetheless, depending on the application, the use of a given CRISPR-Cas system for biotechnology or medicine may need to be assessed for CRISPR crosstalk.

With our study, we did not find a relationship between CRISPR crosstalk and human genomic DNA damage reported in the literature. The mechanism for Cas9-mediated genome-wide DNA damage in human cells in the absence of gRNAs remains a critical open question for the field. Whether the phenomenon be induced by Cas9 translational stress, gRNA-independent Cas9 DNA helicase and/or cleavage activity, or perhaps some as of yet uncharacterized Cas9 modality, it is a problem worth pursuing.

## Methods

### Tissue culture

HEK 293T cells (Takara Bio Lenti-X 293T, #632180) were maintained in DMEM (4.5 g/L D-glucose) supplemented with 10% FBS (Gibco) at 37 °C with 5% CO_2_. Cells were periodically passaged once at 70-90% confluency by dissociating with TrypLE Express Enzyme (Gibco) at a ratio of 1:10.

### Plasmid construction

Protein-expressing plasmids were constructed from *pCDNA3.1(-)* (ThermoFisher Scientific) by Gibson cloning a protein with upstream Kozak and start codon sequences and downstream stop codon sequence into its EcoRI and BamHI restriction enzyme sites. Catalytically inactive dSpCas9 without an NLS was subcloned from Nelles et al.^28^. Catalytically dead SpCas9 with an NLS was subcloned from lentiCRISPR v2 (AddGene #52961). A V5 peptide sequence with G linker (5′-GGCAAACCGATCCCGAATCCGCTTCTTGGTCTTGACTCCACGGGG-3′) was cloned upstream of the expressed protein in *pCDNA3.1-V5-dSpCas9* and *pCDNA3.1-V5-SpCas9-NLS*. A 3xFLAG peptide sequence with G linker (5′- GACTACAAAGACCATGACGGTGATTATAAAGATCATGACATCGACTACAAGGATGACGATGACAAGGGG -3′) was cloned upstream of the expressed protein in *pCDNA3.1-3xFLAG-dSpCas9*. The UnaG protein sequence (Fluorescent Protein Database) was human codon optimized with IDT’s codon optimization tool prior to ordering as a gBlock to clone into *pCDNA3.1-UnaG*. For U6 promoter and U6 promoter-driven gRNAs conditions, the U6 promoter sequence 5′-AGGTCGGGCAGGAAGAGGGCCTATTTCCCATGATTCCTTCATATTTGCATATACGATACAAGGCTGTTAGAGAGATAATTAGAATTAATTTGACTGTAAACACAAAGATATTAGTACAAAATACGTGAC GTAGAAAGTAATAATTTCTTGGGTAGTTTGCAGTTTTAAAATTATGTTTTAAAATGGACTATCATATGCTTACCGTAACTTGAAAGTATTTCGATTTCTTGGCTTTATATATCTTGTGGAAAGGACGAAACACC-3′ with a 5′-TTTTTT-3′ terminator sequence was cloned into *pCDNA3.1-V5-dSpCas9*. The gRNA backbone sequence used was 5′-GTTTAAGAGCTATGCTGGAAACAGCATAGCAAGTTTAAATAAGGCTAGTCCGTTATCAACTTGAAAAAGTGGCACCGAGTCGGTGCT-3′. Sequences for guides 1 and 2 were 5′-GAGTGTCAGCCAGTATAACCC-3′ and 5′-GGCGCGGGCCGCTCGCTCTA-3′, respectively.

### eCLIP experiment

HEK 293T cells were transfected at 60–80% confluence in 10 cm plates using the jetOPTIMUS transfection kit (Polyplus Transfection) with either *pCDNA3.1-V5-dSpCas9, pCDNA3.1-3xFLAG-dSpCas9*, or *pCDNA3.1(-)*. Forty-eight hours post-transfection, biological replicates of confluent 10 cm plates of HEK 293T cells were treated with 400 mJ/cm^2^ of UV using the Stratalinker 2400, harvested in ice cold PBS and pellets flash frozen in liquid nitrogen and stored in −80 °C until ready to IP with either V5 Tag mouse monoclonal antibody (ThermoFisher Scientific #R960-25) or mouse monoclonal ANTI-FLAG M2 antibody (Sigma-Aldrich #F1804) each at a dilution of 1:3000 in a subsequent protocol exactly as detailed in Van Nostrand et al.^6^, cutting the nitrocellulose membrane from 115 kDa and up. The size-matched input not subjected to IP was cut from the identical region. Sequencing was performed on Illumina HiSeq 4000 with paired end reads.

### eCLIP computational analysis

Data were processed through Dr. Yeo’s eCLIP pipeline version 0.4.0 (https://github.com/YeoLab), aligning reads to the human reference genome hg38. Reproducible peaks were assigned using IDR (Irreproducible Discovery Rate) in which entropy was used to rank replicate peaks, and further evaluated using self-consistency and rescue ratios according to Van Nostrand et al. (2016), where detailed information regarding peak calling used for Cas9 in this study can be found. Bed files representing each of the four sets of eCLIP peak IDR analyses (two for V5 and two for FLAG) were intersected with minimum 50% overlap, intersecting the V5 and FLAG eCLIP peaks separately at first. Maximum eCLIP peak enrichment per gene was taken from the V5 vs. size-matched input or FLAG vs. size-matched input IDR analysis, with *R*^2^ statistics performed on these values. Gene regions for each eCLIP peak were determined based on all gene regions represented across all four IDR peaks whose intersection yielded that peak. For the gene region analysis of top three represented regions (5′ UTR, CDS, 3′ UTR), region lengths were normalized over average TPM (transcripts per million) across all four no IP/size-matched input eCLIP RNA-sequencing datasets (two for V5-dSpCas9; two for 3xFLAG-dSpCas9) for genes with TPM > 1. Paired nucleotide probabilities (Vienna RNAplfold default parameters, *u* = 1) were region length- and TPM-normalized for genes with TPM > 1.

### Immunofluorescence (IF) imaging of Cas9 proteins

HEK 293T cells were transfected at 60–80% confluence in Nunc Lab-Tek II Chamber Slides (ThermoFisher Scientific) using the jetOPTIMUS transfection kit (Polyplus Transfection) with either *pCDNA3.1-V5-dSpCas9* or *pCDNA3.1-3xFLAG-dSpCas9*. Slides were fixed with MeOH, blocked for 1 h at room temperature, and incubated under gentle orbital shaking with primary antibody overnight: either V5 Tag mouse monoclonal antibody (ThermoFisher Scientific #R960-25) at 1:3000 dilution or mouse monoclonal ANTI-FLAG M2 antibody (Sigma-Aldrich #F1804) at 1:1000 dilution. Slides were washed five times for 10 min with phosphate-buffered saline with Tween 20 (PBST), then incubated for 1 h at room temperature under gentle orbital shaking with secondary antibody: Goat anti-mouse IgG AlexaFluor 488 Superclonal Recombinant Secondary antibody (ThermoFisher Scientific #A28175) at 1:2000 dilution. Slides were washed five times for 10 min with PBST, then washed three more times with PBS before mounting overnight with 4',6-diamidino-2-phenylindole (DAPI). All antibodies were incubated with 5% BSA in 0.1% Tween-PBS. Immunofluorescence images were taken at 63x objective with a Zeiss LSM 780 confocal microscope in 5–10 slices, with maximum intensity projections across the entire image plane generated in Zeiss ZEN 2010 for figures.

### Gene ontology enrichment of eCLIP genes

Gene ontology enrichment was performed on the 381 eCLIP genes with Panther, using a background of 11,405 genes derived from size-matched inputs (average TPM > 1 among 1N, 4N, 6N, 2N).

### Computational analysis of stress pathway-associated eCLIP genes

eCLIP genes associated with the Panther GO gene ontology accession GO:0033554 cellular response to stress (*n* = 66) were selected. For each gene, the mean of its log2 RPKM L1000 expression over 165 human cancer cell lines was taken from a Cas9 gene expression dataset in Enache et al.^10^, filtering out those with <1 log2 RPKM L1000 expression in any of the 165 control cell lines (*n* = 55 genes).

### RT-qPCR of stress pathway-associated eCLIP genes

HEK 293T cells were transfected at 60–80% confluence in six-well plates using the jetOPTIMUS transfection kit (Polyplus Transfection) with either *pCDNA3.1-V5-dSpCas9, pCDNA3.1-V5-dSpCas9-U6 promoter, pCDNA3.1-V5-dSpCas9-U6-gRNA 1, pCDNA3.1-V5-dSpCas9-U6 gRNA 2, pCDNA3.1-V5-SpCas9-NLS, pCDNA3.1(-)*, or *pCDNA3.1-UnaG* in three bioreplicates per condition. RNA was extracted from cells with the RNeasy Plus kit (Qiagen). Approximately, 1 µg of RNA was converted into cDNA with the ProtoScript II First Strand cDNA Synthesis kit (NEB) with random primers. qPCR for two technical replicates of each of the three bioreplicates with a distinct pair of PCR primers per gene was performed on a CFX384 Touch Real-Time PCR Detection System (Bio-Rad) with 1/6 diluted cDNA samples at 2 µL input in PowerTrack SYBR Green Master Mix (ThermoFisher Scientific), for 95 °C initial incubation for 2 min, followed by 40 cycles of 95 °C for 15 s and 60 °C for 1 min. Technical replicates were averaged for each of the three bioreplicates per condition. In analysis each gene’s expression was compared to GAPDH housekeeping gene expression to compute Δct values. Then *–*ΔΔct values were computed for each condition-bioreplicate-gene Δct with respect to the mean gene Δct of the *pCDNA3.1(-)* bioreplicates. Comparisons among a given gene’s condition-bioreplicate –ΔΔct values were made pairwise with one-way ANOVA. PCR primer pairs for given genes are as follows: *GAPDH* (F: 5′-GTCTCCTCTGACTTCAACAGCG-3′, R: 5′-ACCACCCTGTTGCTGTAGCCAA-3′); *ACTB* (F: 5′-CACCATTGGCAATGAGCGGTTC-3′, R: 5′-AGGTCTTTGCGGATGTCCACGT-3′); *p53* (F: 5′-GAGCTGAATGAGGCCTTGGA-3′, R: 5′-CTGAGTCAGGCCCTTCTGTCTT-3′); *CDIP1* (F: 5′-ATTGGCTTGATGAATTTCGTGC-3′, R: 5′-GTGCGTCACATCCTTGAAGTC-3′); *ATF3* (F: 5′-CCTCTGCGCTGGAATCAGTC-3′, R: 5′-TTCTTTCTCGTCGCCTCTTTTT-3′); *CDKN1A (p21)* (F: 5′-AGGTGGACCTGGAGACTCTCAG-3′, R: 5′-TCCTCTTGGAGAAGATCAGCCG-3′).

### Western blots

Frozen pellets containing 10 million cells were recovered from −80 °C. Protease inhibitor III (Millipore Sigma #539134) was combined with iCLIP lysis buffer (50 mM Tris-HCL pH 7.4, 100 mM NaCl, 1% NP-40 Igepal CA630, 0.1% SDS, 0.5% Sodium deoxycholate). Cells were lysed with eCLIP lysis buffer and protease inhibitor for 15 min on ice and then sonicated on low for 5 min, 30 s on/30 s off. Lysed cells were centrifuged at 15,000 × *g* for 4 min. The supernatant was aliquoted into 100 µL aliquots to be stored at −80 °C to prevent protein degradation. Protein concentration was measured by Pierce BCA Protein Assay (ThermoFisher Scientific #23227). Fifty micrograms of protein was run on NuPAGE 4–12% Bis-Tris Gel (ThermoFisher Scientific #NP0335BOX) at 150 V for 1.5 h. Gels were transferred via iBlot 2 Gel Transfer Device (ThermoFisher Scientific #IB21001), blocked in 5% milk, and put in primary overnight. Florescent antibodies were utilized for multiplexing. For ATF3, primary antibody Recombinant Anti-ATF3 antibody [EPR22610-19] (Abcam #ab254268) at 1:1000 dilution and secondary antibody IRDye 680RD Goat anti-Rabbit IgG Secondary Antibody (Li-Cor #926-68071) at 1:20,000 dilution were used. For alpha tubulin, primary antibody Anti-alpha Tubulin antibody [DM1A] - Loading Control (Abcam **#**ab7291) at 1:5000 dilution and secondary antibody IRDye 800CW Goat anti-Mouse IgG Secondary Antibody (Li-Cor #926-32210) at 1:20,000 dilution were used. Membranes were visualized using the Azure biosystems c600. Proteins were quantified using ImageJ Version 2.0.0-rc-69/1.52n (https://imagej.nih.gov/).

### Electrophoretic mobility shift assays (EMSAs)

All EMSAs were performed with SpCas9-NLS protein (CAS9PROT from Sigma-Aldrich) in EMSA buffer (20 mM Tris-HCL pH 7.4, 150 mM KCl, 5 mM MgCl_2_, 0.1% BSA, 1 mM DTT, 5 mM EDTA, 200 U/mL Superase-In RNase Inhibitor (ThermoFisher Scientific), 5% glycerol, 0.01% Tween 20, 50 µg/mL heparin). RNA was in vitro transcribed with the MEGAscript T7 Transcription kit (ThermoFisher Scientific) and purified with RNA Clean & Concentrator-5 (Zymo). Labeled RNA was 5′ labeled with the 5′ EndTag Labeling DNA/RNA kit (Vector Laboratories) and IRDye 800CW Maleimide (Li-COR Biosciences). After incubating protein and RNA in EMSA buffer for 30 min at room temperature, 10x Orange loading dye (Li-COR Biosciences) was added to samples before pipetting into gels pre-run for 20 min at 120 volts at 4 °C. Gels were resolved by running for 1 h at 120 volts at 4 °C on 6% Novex TBE gels (ThermoFisher Scientific) with 0.5x TBE buffer. Images were taken with the Azure Biosystems c600 imager.

#### Competitive EMSA

Cas9 protein at 640 nM was incubated with 20 nM 5′-end fluorescently labeled in vitro transcribed *CDIP1 5*′ *UTR RNA* (5′-UACCCGCCUCCUUGUGACAGAAGUGCGACUGCCAGCUGCCGAGGC**GU**UCGGUCCUGCUGUUGCGGCCGCUGCCCCAGGGCUGCGGGGACGGUGAGUCGACUGGA-3′) and either unlabeled in vitro transcribed *Cas9 gRNA* (5′-AUUAAUCGGUGGGAGUAUUCGUUUAAGAGCUAUGCUGGAAACAGCAUAGCAAGUUUAAAUAAGGCUAGUCCGUUAUCAACUUGAAAAAGUGGCACCGAGUCGGUGCU-3′) or unlabeled in vitro transcribed non-specific *N.S. RNA* (5′-CUAUGCGGCAUCAGAGCAGAUUGUACUGAGAGUGCACCAUAUGCGGUGUGAAAUACCGCACAGAUGCGUAAGGAGAAAAUACCGCAUCAGGCGCCAUUCGCCAUUCAGGCUGCGCAACUGUUGG-3′) at molar ratios of 1:1 to 1:32 with respect to Cas9 protein.

#### Mutant CDIP1 EMSAs (Fig. 2)

Cas9 protein at 0, 198, 296, 444, 667, and 1000 nM was incubated with 20 nM 5′-end fluorescently labeled in vitro transcribed *N.S. RNA, CDIP1 5*′ *UTR RNA, CDIP1 5*′ *UTR RNA (loop G* > *U)*, or *CDIP1 5*′ *UTR RNA (loop U* > *A)*. GU-loop sequence is emboldened and underlined in the *CDIP1 5*′ *UTR RNA* sequence above.

#### Mutant CDIP1 EMSAs (Supplementary Fig. 6)

In vitro transcribed RNAs were 3′ end labeled with Terminal Deoxynucleotidyl Transferase (ThermoFisher Scientific) and Propargylamino-dCTP-Cy5 (Sigma-Aldrich). Cas9 protein at 0, 148, 222, 333, 500, and 750 nM was incubated with 5 nM 3′ end fluorescently labeled in vitro transcribed *CDIP1 5*′ *UTR RNA* and various GU-loop and 5nt-stem mutants depicted in the figure.

Relevant uncropped EMSA gels can be found in Supplementary Figs. 11 and 12.

### In silico RNA secondary structure modeling

The minimum free-energy secondary structure of CDIP1 5′ UTR RNA was predicted in *RNAfold* (Vienna RNA Websuite). A model for SpCas9 RNA binding to eCLIP peaks was developed with *RNAplfold* (Vienna RNA Websuite), based on the GU-loop:5nt-stem of SpCas9 in complex with its gRNA (base-pairing probability of loop *U* < 0.7; base-pairing probability of each of the five stem bases > 0.5), under default parameters with 50 nt padding on either side of an input RNA sequence (unspliced, for consistency given that some peaks are located on unspliced RNA). The prediction performance of this model was compared for eCLIP peak sequences (*n* = 478) and eCLIP genes with peak sequences (*n* = 381) against 1000 Monte Carlo simulations of random shuffles of the eCLIP peak sequences (*n* = 478).

### In vivo RNA secondary structure modeling

In vivo click selective 2-hydroxyl acylation and profiling experiment (icSHAPE) structure probing data of HEK 293T transcripts^18^ were utilized. For inclusion in the analysis, Cas9-interacting RNA transcripts were required to have (i) an eCLIP peak represented by two valued replicates of icSHAPE reactivities across the entire eCLIP peak, (ii) only one eCLIP peak per transcript, and (iii) a peak interval length of at least 50nt. This quality control filter yielded a total of ten eCLIP peaks. Experimental folds were computed using RNApvmin and RNAfold (Vienna RNA Websuite; Mathews et al.^20^; Deigan et al.^19^ parameters of slope 1.9 and intercept −0.7).

### Computational analysis of RNA-Seq data for nickase Cas9-APOBEC with gRNA co-expressed in HEK 293T cells

Editing sites with edit rates for replicates 1 and 2 of EMX, RNF2, and N.T. gRNA were taken from Supplementary Tables 11, 12, and 13 of Grünewald et al.^25^ For each of the six replicates, the total number of editing sites per gene (as determined by alignment to GENCODE v29) was plotted in a box plot for non-eCLIP genes alongside eCLIP genes. Only genes with at least one editing site were plotted for each cohort. On a per gene basis C-to-U edit site counts were compared to the average TPM (transcripts per million) across all four no IP/size-matched input eCLIP RNA-sequencing datasets (two for V5-dSpCas9; two for 3xFLAG-dSpCas9), with *R*^2^ statistics performed on these values. The mean fraction of edits within W (50, 100, 200, 500) nt distance of eCLIP peaks was calculated for each unique eCLIP peak whose midpoint mapped to spliced RNA. Briefly, for each eCLIP peak midpoint, the fraction of all C-to-U edit sites on its spliced transcript within W nt distance was calculated. For each of the six replicates, the mean of this value over all eCLIP peaks was then calculated. For the 10,000 Monte Carlo simulations, simulated eCLIP peaks were placed according to a uniform random distribution across their respective spliced RNA transcripts.

### Computational analysis of CHIP-Seq data for catalytically dead Cas9 with gRNA co-expressed in HEK 293T cells

CHIP-Seq data for replicates 1 and 2 of gRNAs 1, 2, and 3 were taken from GEO: GSE55887 of Kuscu et al.^22^ Reads were mapped to the human reference genome hg38 and converted to bedgraph file form using bowtie (1.2.2) and bedtools (2.27.1) with read coverage normalized to reads per million. For each of the six replicates, the maximum single-base read coverage per gene (as determined by alignment to GENCODE v29) was plotted in a box plot for non-eCLIP genes alongside eCLIP genes. For inclusion in a cohort, genes were required to have at least one mapped read in the CHIP-Seq dataset and TPM > 1, where TPM are the average transcripts per million across all four no IP/size-matched input eCLIP RNA-sequencing datasets (two for V5-dSpCas9; two for 3xFLAG-dSpCas9). On a per gene basis maximum single-base read coverages were compared to the average TPM across all four no IP/size-matched input eCLIP RNA-sequencing datasets (two for V5-dSpCas9; two for 3xFLAG-dSpCas9), with *R*^2^ statistics performed on these values.

### Computational analysis of GUIDE-Seq data for Cas9 with gRNA co-expressed in HEK 293T cells

GUIDE-Seq data for the *no Cas/no gRNA* negative control and gRNAs 1, 2, 3, and 4 were taken from SRA: SRP050338 of Tsai et al.^21^ Reads were processed into unique reads from UMIs and then mapped to the human reference genome hg38 using the GUIDE-Seq pipeline (https://github.com/tsailabSJ/guideseq) and BWA (0.7.17), with reads normalized to reads per million. For each of the five conditions, the total number of mapped reads per gene (as determined by alignment to GENCODE v29) was plotted in a box plot for non-eCLIP genes alongside eCLIP genes. Only genes with at least one mapped read were plotted for each cohort.

### General computational analysis

Custom scripts written in Python 3.7.7 and MATLAB 2019b were used to analyze and plot data.

### Reporting summary

Further information on research design is available in the Nature Research Reporting Summary linked to this article.

## Supplementary information

Supplementary Information

Reporting Summary

Description of Additional Supplementary Files

Supplementary Data 1

Supplementary Data 2

## Data Availability

The sequencing data generated in this study have been deposited in the NCBI GEO (Gene Expression Omnibus) database under accession code GSE167466. All uncropped EMSA and Western blot gel image files critical to the manuscript have been made available in the Supplementary Information.
