# Supplementary material for: Crosstalk between CRISPR-Cas9 and the human transcriptome
Source: Nat Commun. 2022 Mar 2;13:1125. doi: 10.1038/s41467-022-28719-5 (PMC8891275; doi:10.1038/s41467-022-28719-5)
Supplement: Supplementary file 1 — Supplementary Information [file 41467_2022_28719_MOESM1_ESM.pdf]

Supplementary Information for

Crosstalk between CRISPR-Cas9 and the human transcriptome

Aaron A. Smargon[1,2,3], Assael A. Madrigal[1,2,3], Brian A. Yee[1,2,3], Kevin D. Dong[1,2,3], Jasmine R. Mueller[1,2,3], Gene W. Yeo[1,2,3,*]

[1] Department of Cellular and Molecular Medicine, University of California San Diego, 9500 Gilman Drive, La Jolla, CA 92093 USA

[2] Stem Cell Program, University of California San Diego, Sanford Consortium for Regenerative Medicine, 2880 Torrey Pines Scenic Drive, La Jolla, CA 92037 USA

[3] Institute for Genomic Medicine, University of California San Diego, 9500 Gilman Drive, La Jolla, CA 92093 USA

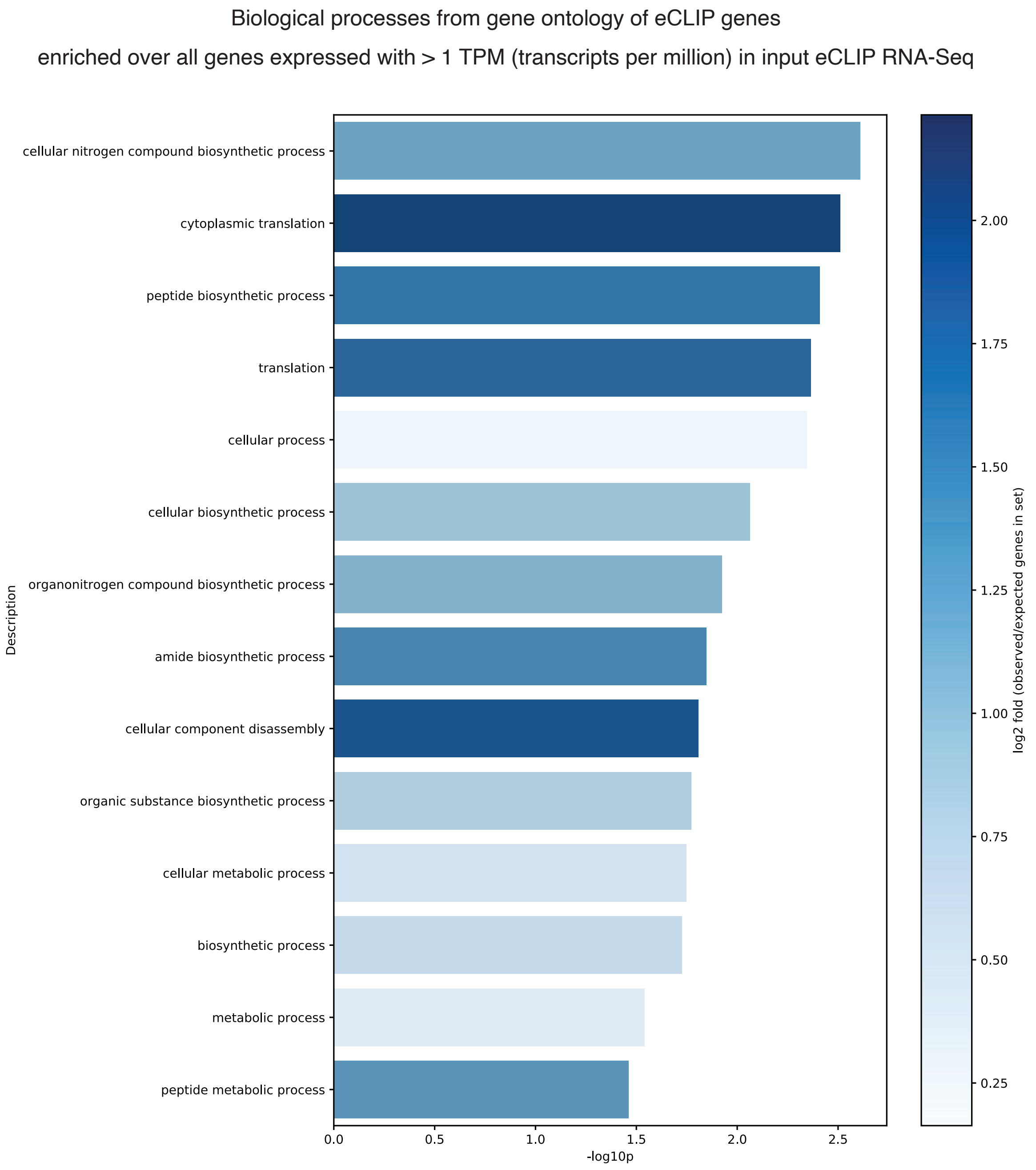

Supplementary Fig. 1. Biological processes enriched in eCLIP genes. Biological processes from gene ontology of eCLIP genes enriched over all genes expressed with > 1 TPM (transcripts per million) in input eCLIP RNA-sequencing.

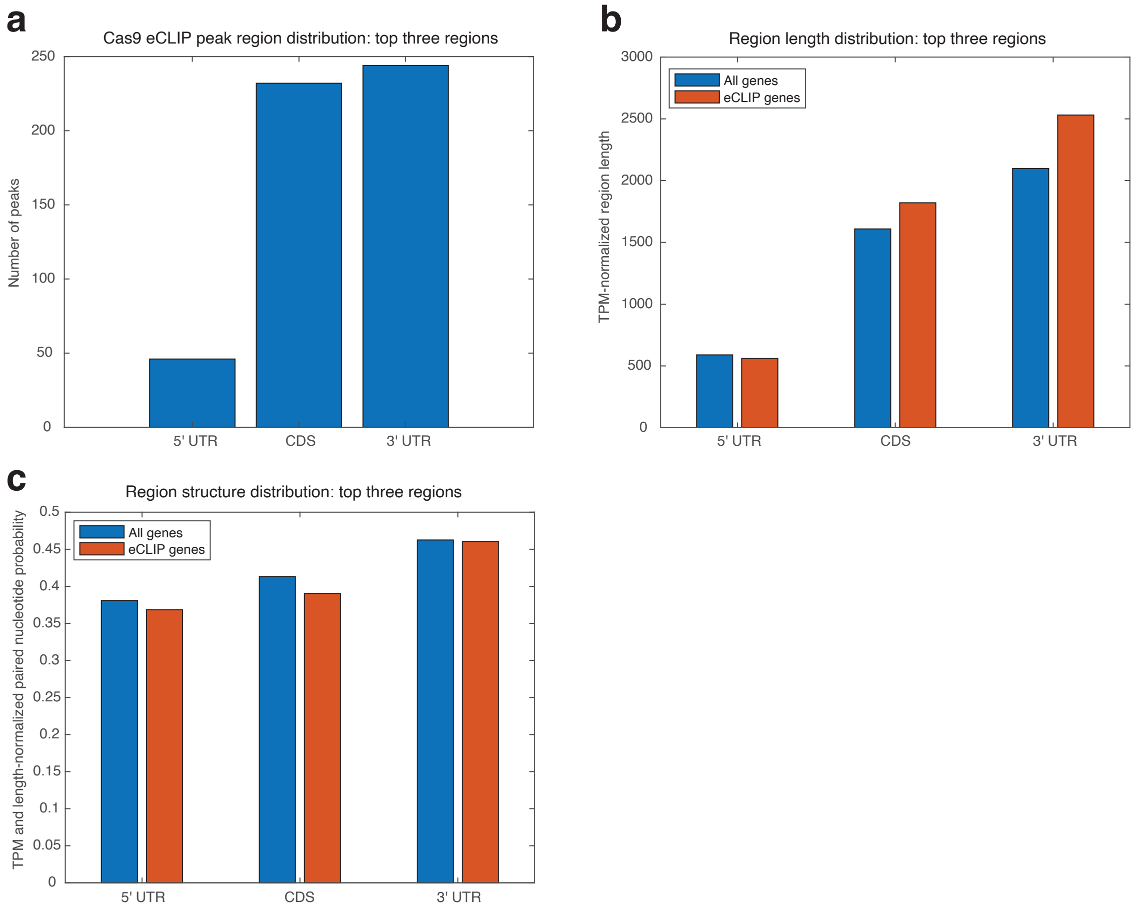

Supplementary Fig. 2. The eCLIP peak region distribution of the top represented gene regions correlates with region length. a, Bar plot of the number of peaks represented in each of the top three gene regions (5′ UTR, CDS, 3′ UTR) among Cas9 eCLIP peaks. **b,** Bar plot of the TPM (transcripts per million)-normalized region lengths in the input eCLIP RNA-sequencing across genes with TPM > 1 for each of the top three gene regions (5′ UTR, CDS, 3′ UTR): all genes vs. eCLIP genes. **c,** Bar plot of the TPM (transcripts per million)-normalized in the input eCLIP RNA-sequencing across genes with TPM > 1 and region length-normalized paired nucleotide probability according to Vienna RNAplfold for each of the top three gene regions (5′ UTR, CDS, 3′ UTR): all genes vs. eCLIP genes.

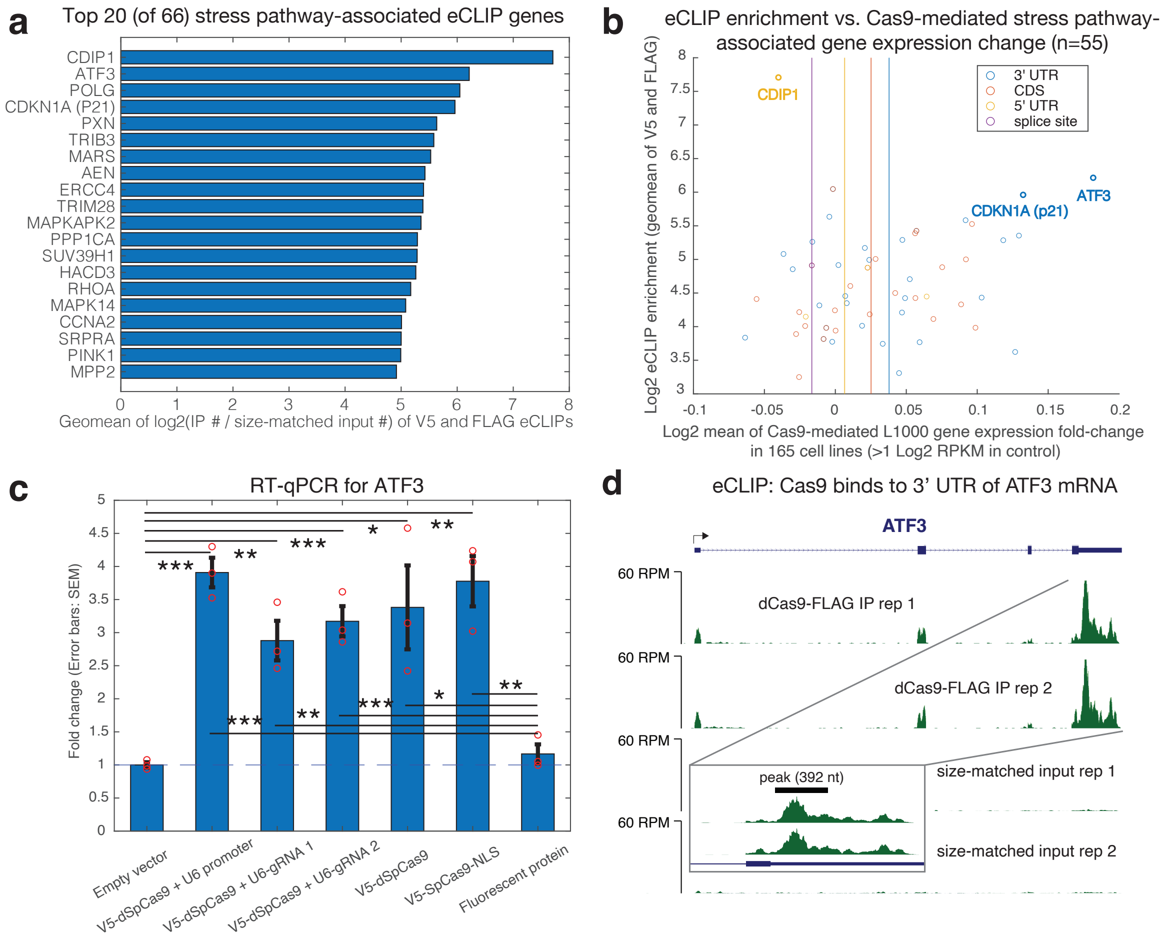

Supplementary Fig. 3. Cas9 interacts with transcripts of cellular stress response pathway-associated human genes. a, Top 20 of the 66 stress response pathway-associated genes with whose transcripts dSpCas9 interacts in eCLIPs. **b,** eCLIP gene enrichment (geomean of V5 and FLAG eCLIPs) vs. mean Cas9-mediated (log2-fold) gene expression changes across 165 human cancer cell lines with > 1 log2 RPKM in the no-Cas9 control condition according to Enache et al. (2020) for stress response pathway-associated Cas9 eCLIP peak genes (n=55). Genes are colored by gene region of eCLIP peaks, with mean region gene expression changes represented by corresponding solid vertical lines. **c,** The mRNA of dSpCas9-interacting ATF3 is differentially upregulated by both dSpCas9 and dSpCas9-NLS, as well as by dSpCas9 co-expressed with U6-driven gRNAs, but not by empty vector or fluorescent protein expression. RT-qPCR was

performed in 3 bioreplicates and 2 technical replicates, with GAPDH as housekeeping gene. Error bars represent standard error of the mean. * $P < 0.05$, ** $P < 0.01$, *** $P < 0.001$ (one-way ANOVA, pairwise comparisons). **d,** dSpCas9 binds to the 3′ UTR of mRNA of the stress response pathway-regulating transcription factor ATF3.

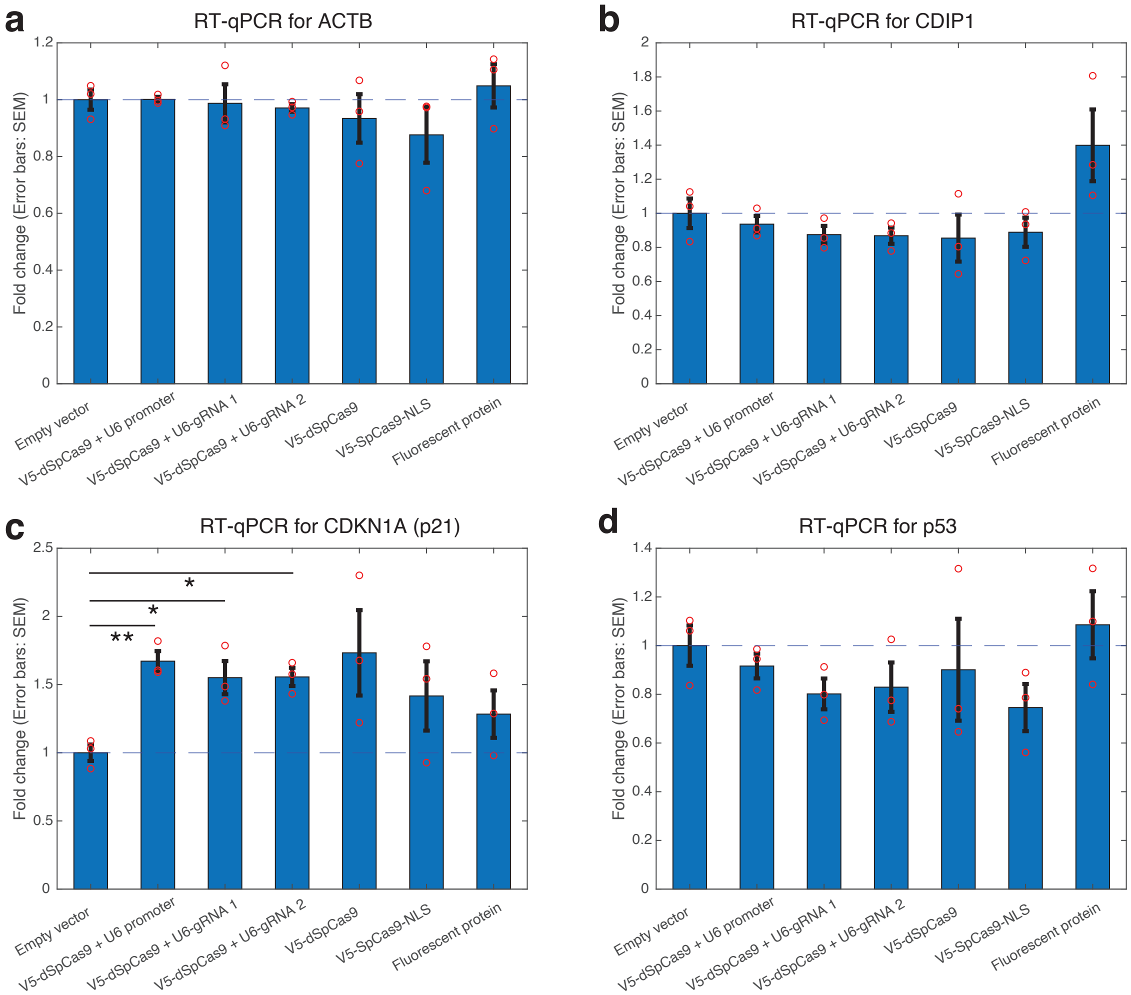

Supplementary Fig. 4. Genes with little or inconsistent RNA level fold change upon Cas9 expression. Expression fold change for mRNA of **a** ACTB, **b** CDIP1**, c** CDNK1A (p21), and **d** p53 under various conditions, including Cas9 with an without U6-driven gRNA. RT-qPCR was performed in 3 bioreplicates and 2 technical replicates, with GAPDH as housekeeping gene. Error bars represent standard error of the mean. * $P < 0.05$, ** $P < 0.01$, *** $P < 0.001$ (one-way ANOVA, pairwise comparisons).

a

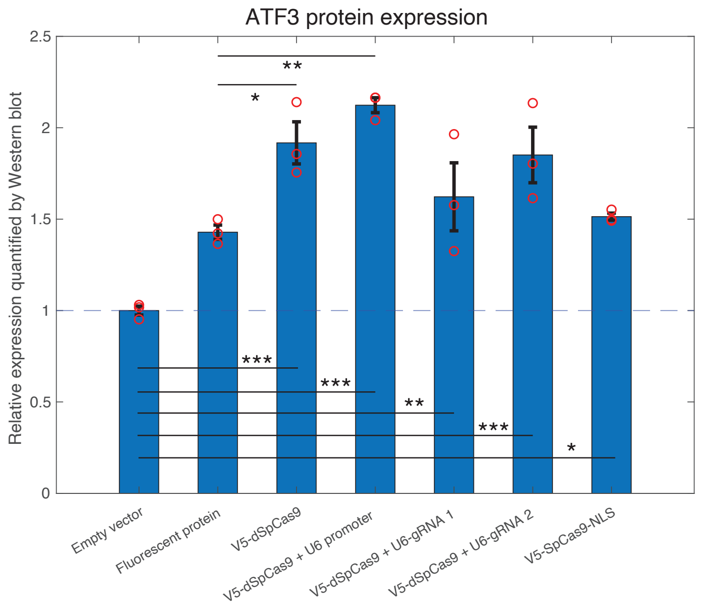

b

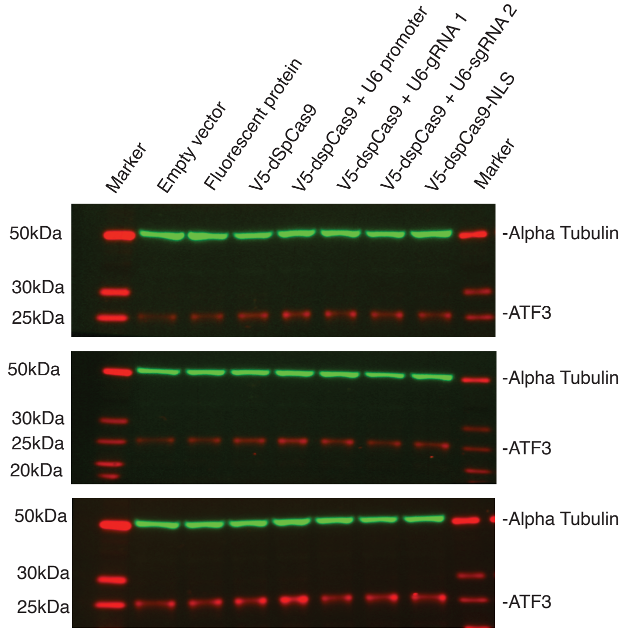

Supplementary Fig. 5. Cytoplasm-localized Cas9 expressed without gRNA upregulates ATF3 protein expression. a, ATF3 protein expression as assessed by Western blot under various conditions, including Cas9 with and without U6-driven gRNA. Western blots were conducted in 3 replicates and quantitated relative to a alpha tubulin control. Error bars represent standard error of the mean. * $P < 0.05$, ** $P < 0.01$, *** $P < 0.001$ (one-way ANOVA, pairwise comparisons). **b,** Western blot gels from which **a** is quantified.

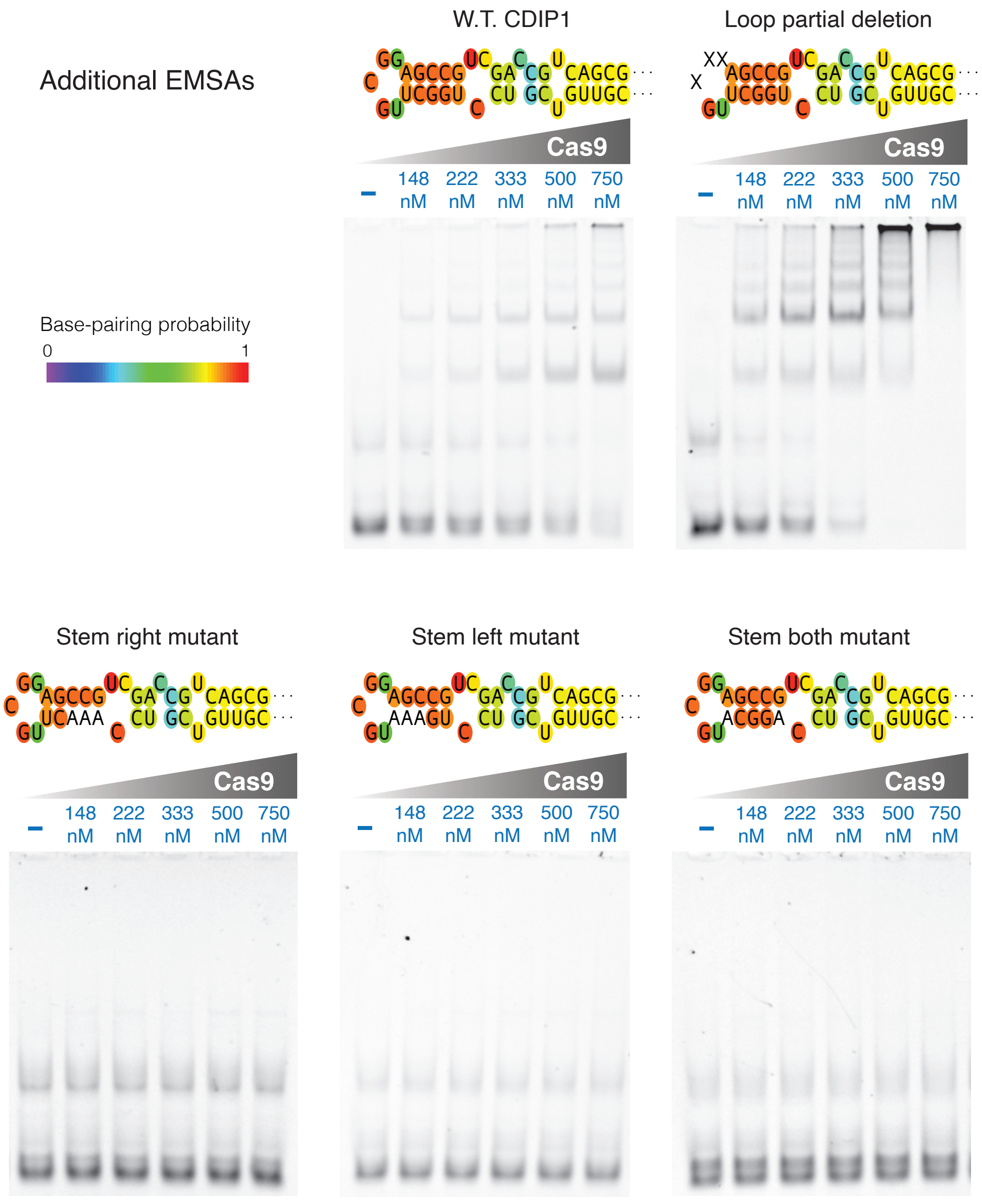

Supplementary Fig. 6. Further mutations to the GU loop or 5nt stem of CDIP1 mRNA reveal Cas9 binding constraints. SpCas9 protein binds to 5nM of 3′-fluorescently labeled CDIP1 mRNA with an apparent dissociation constant (K_D) in the high nanomolar range. A deletion of the stem which does not include the GU motif enhances Cas9 affinity. Mutations to parts of the 5nt stem intended to disrupt RNA secondary structure abolish Cas9 affinity. EMSAs were performed in triplicate, with a representative gel shown.

In vivo HEK 293T icSHAPE (Corley et al., 2020) minimum free energy folds
of quality control filter-passing eCLIP peaks from Cas9-interacting RNA transcripts

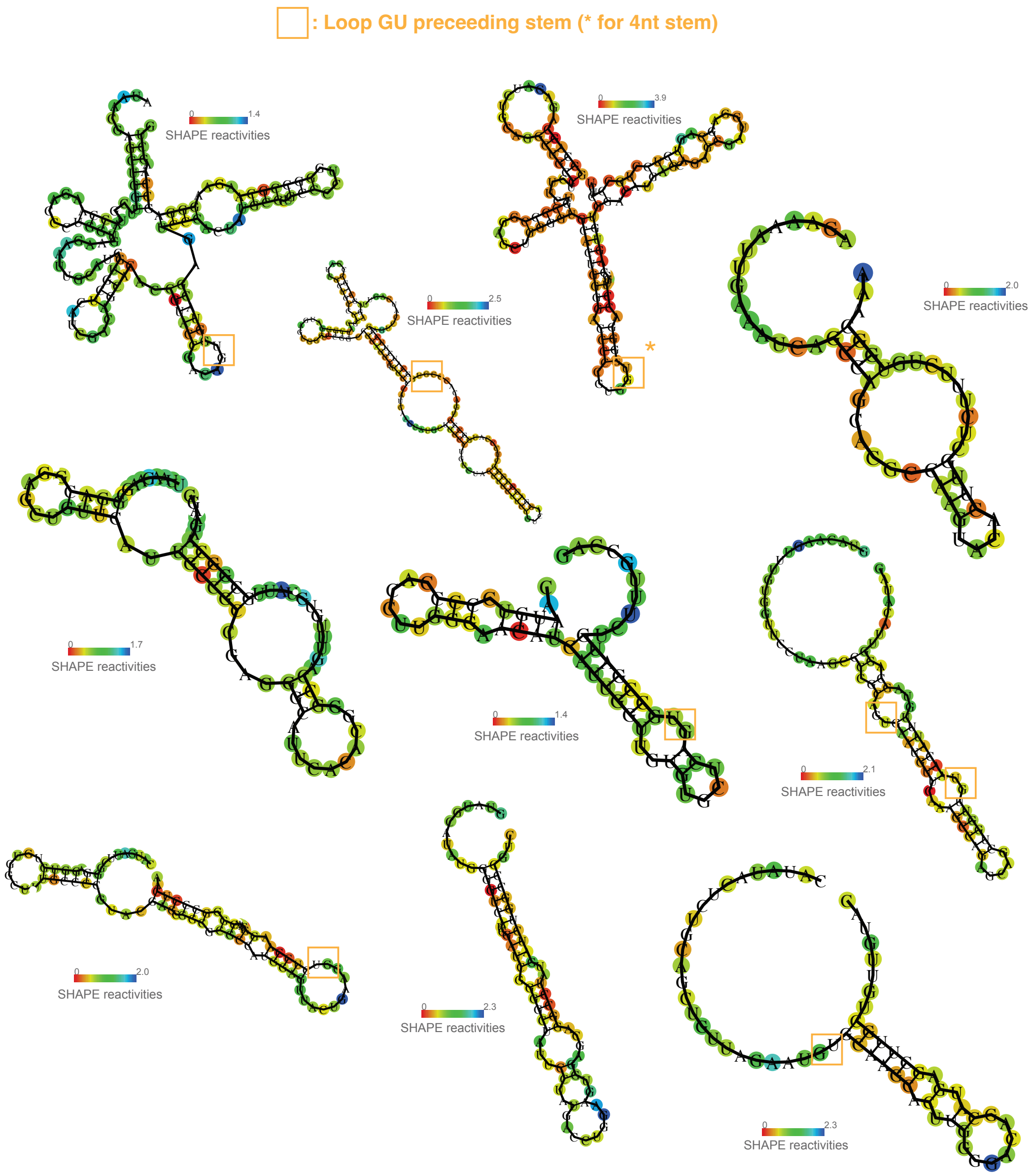

Supplementary Fig. 7. The GU-loop:5nt-stem model extends to Cas9 eCLIP peak RNA structures experimentally derived from human cells. Minimum free energy RNA folds (Vienna RNAfold) based on icSHAPE (in vivo click selective 2-hydroxyl acylation and profiling experiment) RNA structure probing data of *in vivo* HEK 293T transcripts (Corley et al. 2020), from ten Cas9-interacting RNA transcript eCLIP peaks passing a quality control filter (see Methods). Superimposed orange rectangles indicate GU motifs that precede 5nt-stems.

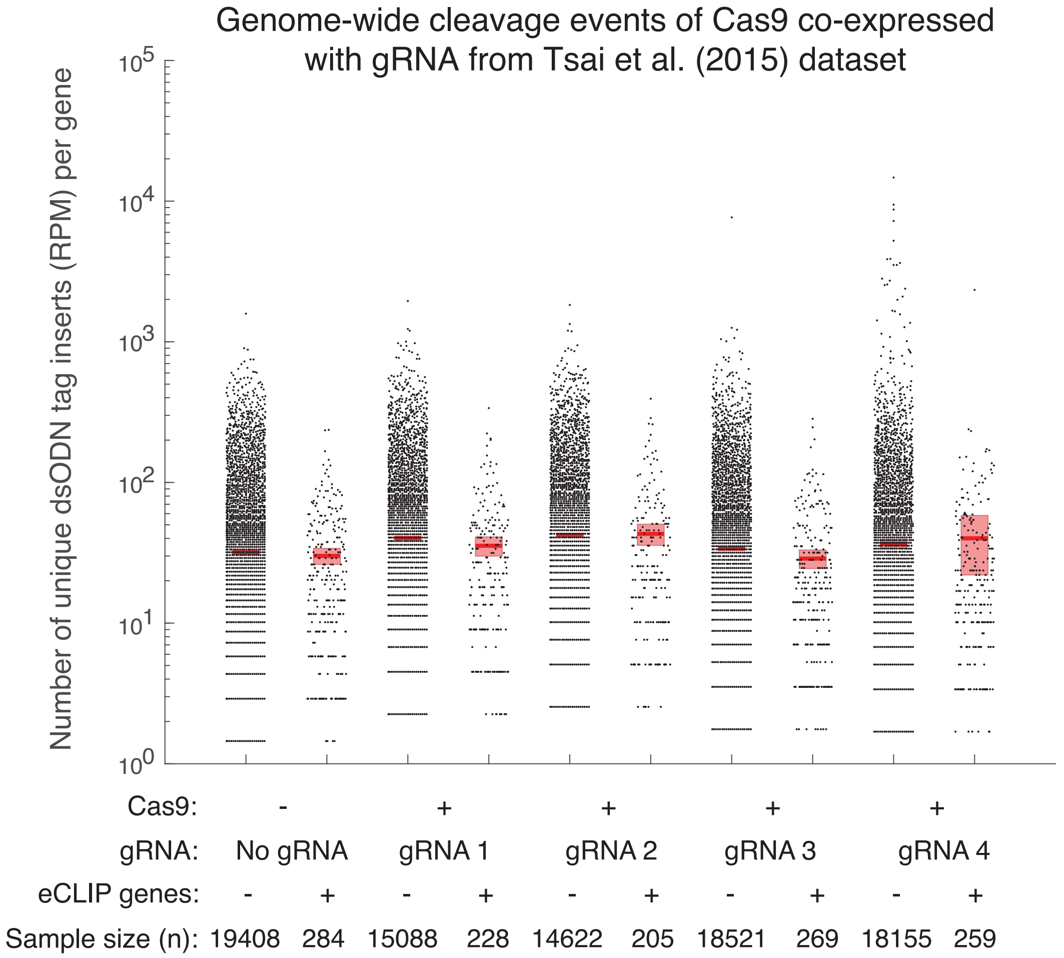

Supplementary Fig. 8. Cas9-interacting human RNA transcripts do not reproducibly correlate with elevated genome-wide DNA editing activities at their target genes under gRNA co-expression. Box plots of the number of unique dsODN tag inserts in reads per million per gene, for non-eCLIP vs. eCLIP genes. Data for each experimental condition (one with no Cas9 or gRNA and four with different gRNAs co-expressed with Cas9) are plotted only of genes with at least one unique mapped dsODN tag insert read according to Tsai et al. (2015). For each dataset mean is bold horizontal red line and 95% confidence interval is lighter red box. (Invisible plotted confidence intervals are within plotted means.)

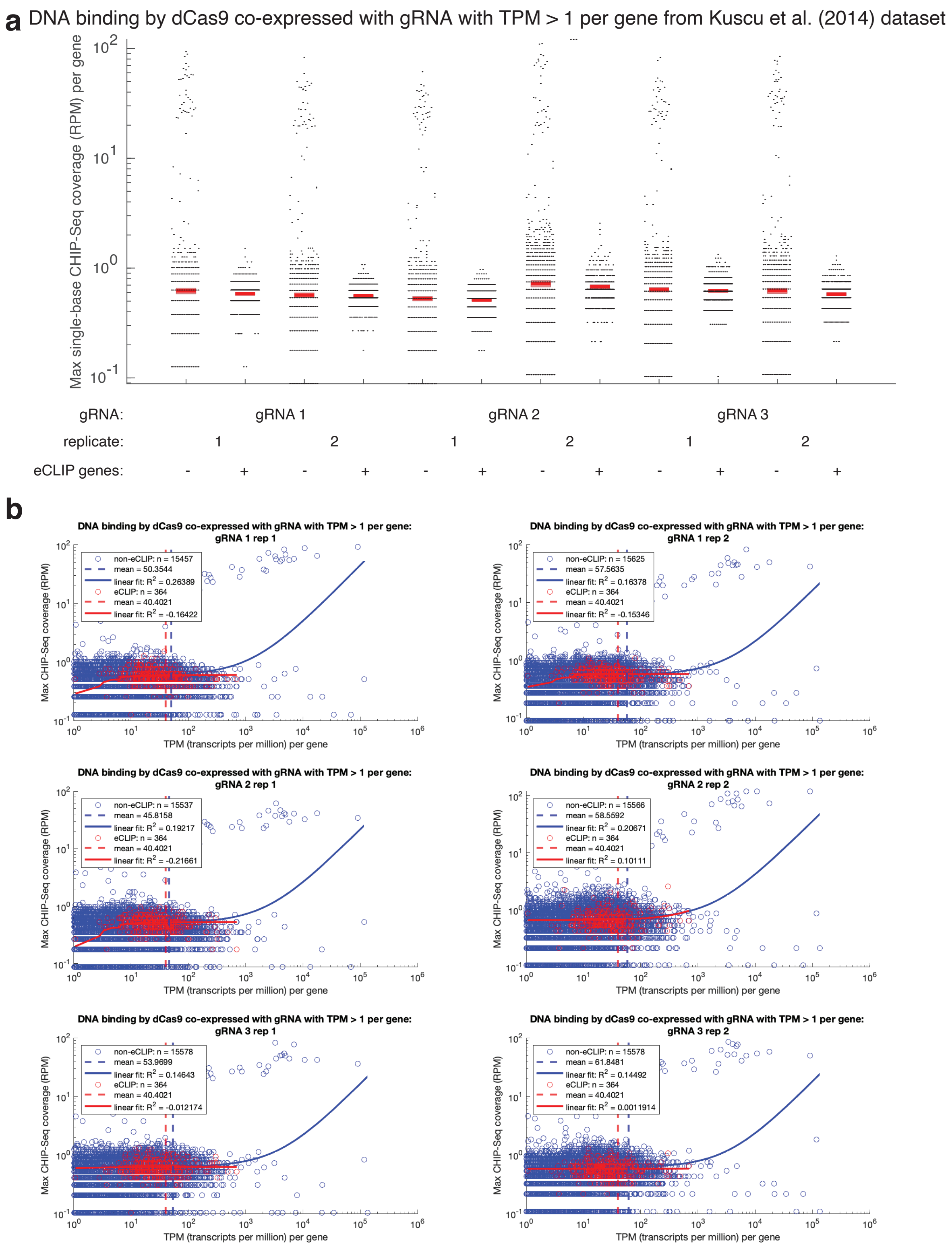

Supplementary Fig. 9. Elevated Cas9 genome-wide DNA binding activities reproducibly correlate with elevated gene expression, but not with Cas9-interacting human RNA

transcript genes under gRNA co-expression. a, Box plots of maximum CHIP-Seq coverage in reads per million per gene of genes with TPM (transcripts per million) > 1 in input eCLIP RNA-sequencing, for non-eCLIP vs. eCLIP genes. Data for each experimental condition (two replicates of three different gRNAs co-expressed with dCas9) are plotted only of genes with at least one mapped read according to Kuscu et al. (2014). For each dataset mean is bold horizontal red line and 95% confidence interval is lighter red box. (Invisible plotted confidence intervals are within plotted means.). **b,** Scatter plot based on **a** of TPM per gene vs. maximum CHIP-Seq coverage in reads per million per gene of genes with TPM > 1 in input eCLIP RNA-sequencing, for non-eCLIP (blue) vs. eCLIP (red) genes. Linear fits with R^2 values of each dataset by gene type are plotted in solid lines, whereas TPM means of each dataset by gene type are plotted in dashed lines.

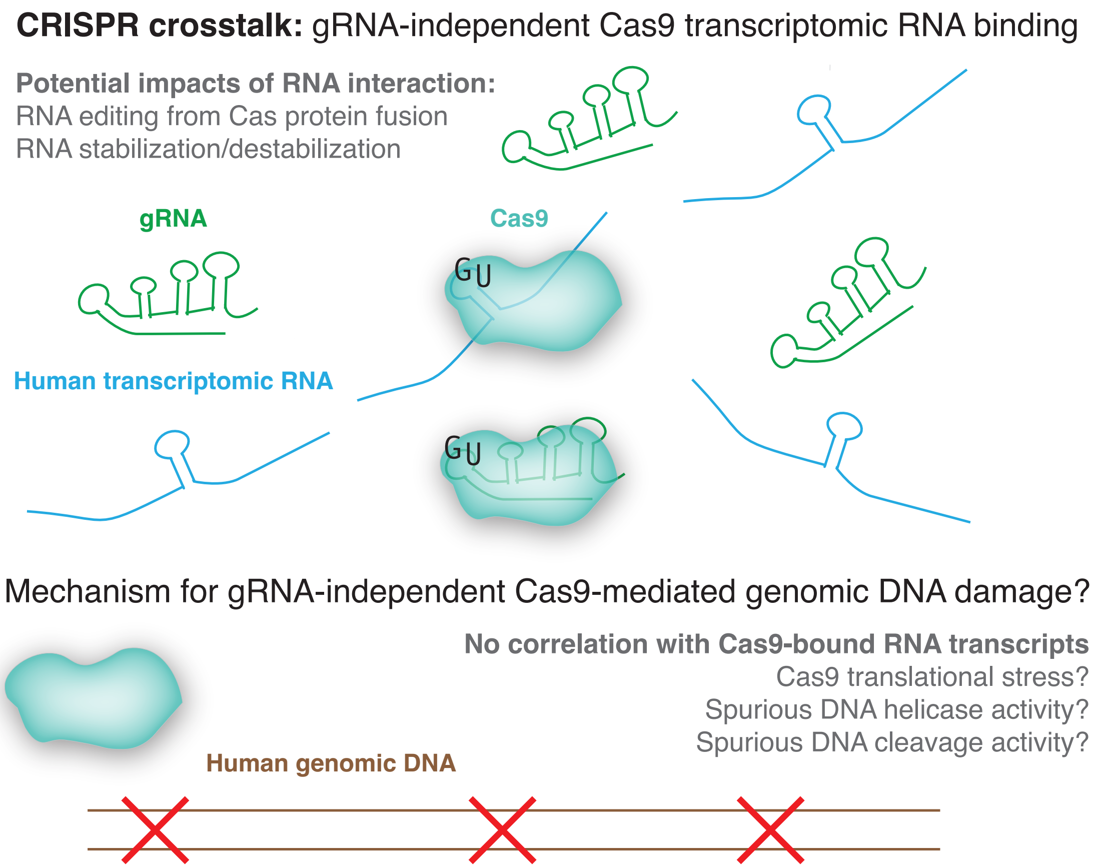

Supplementary Fig. 10. Proposed model for CRISPR crosstalk mechanism. Illustrated diagram for the proposed CRISPR crosstalk mechanism model.

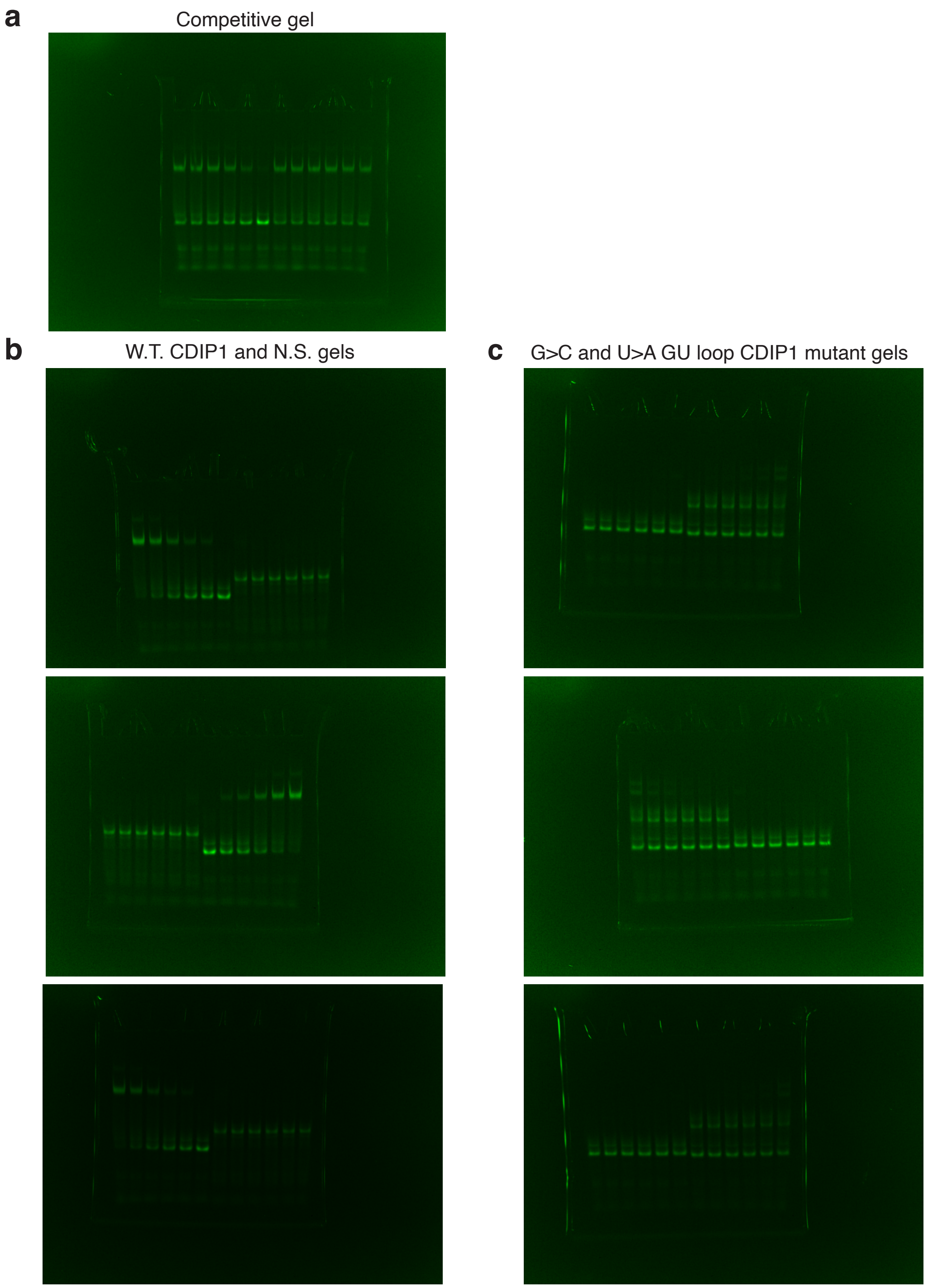

Supplementary Fig. 11. Original EMSA images for data in Fig. 2. a, Raw, non-inverted EMSA gel image files for Fig. 2b. **b,** Raw, non-inverted EMSA gel image files for Fig. 2c (W.T. CDIP1 5′ UTR RNA and N.S. RNA). **c,** Raw, non-inverted EMSA gel image files associated with Fig. 2c (G>C GU-loop CDIP1 5′ UTR RNA and U>A GU-loop CDIP1 5′ UTR RNA).

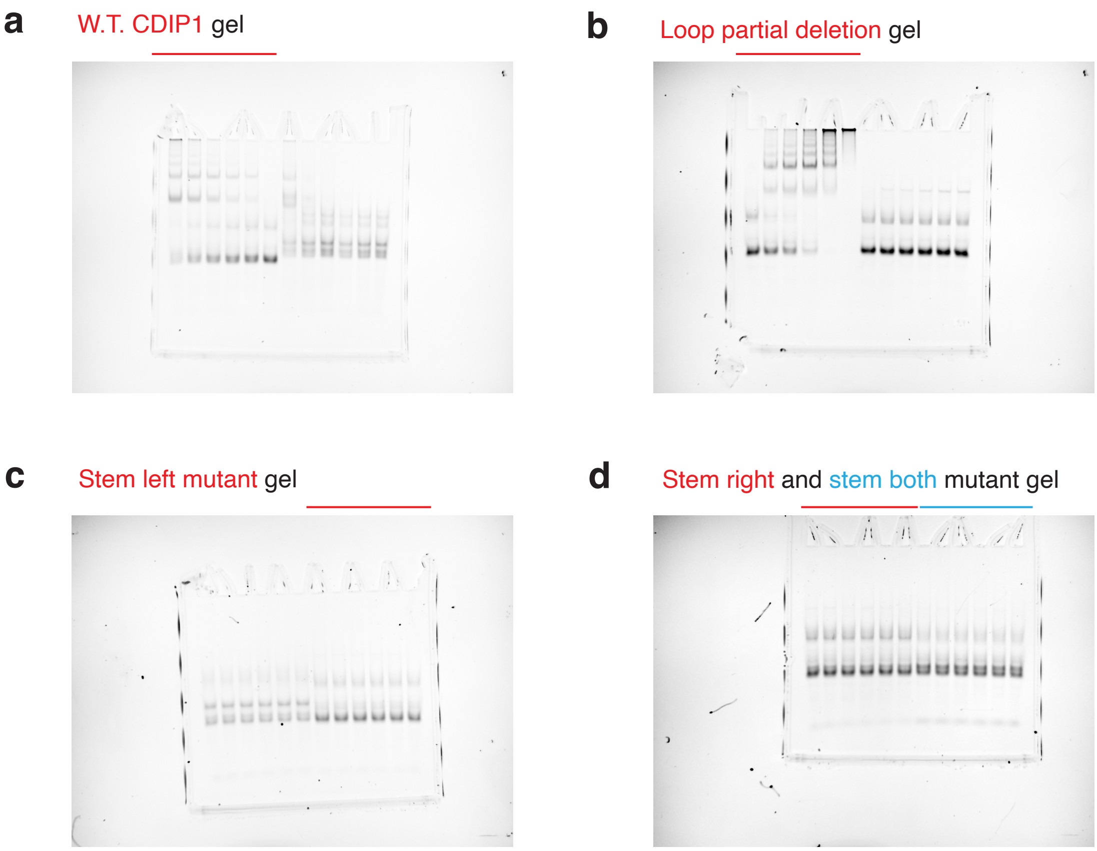

Supplementary Fig. 12. Uncropped EMSA images for data in Supplementary Fig. 6. a, EMSA gel image file for W.T. CDIP1 condition. **b,** EMSA gel image file for loop partial deletion condition. **c,** EMSA gel image file for stem left mutant condition. **d,** EMSA gel image file for stem right mutant and stem both mutant conditions. **(**For subpanels **a-d**, red and blue horizontal lines correspond to respective colored conditions' gel wells.)
