# Supplementary material for: Crosstalk between CRISPR-Cas9 and the human transcriptome
Source: Nat Commun. 2022 Mar 2;13:1125. doi: 10.1038/s41467-022-28719-5 (PMC8891275; doi:10.1038/s41467-022-28719-5)
Supplement: Supplementary file 2 — Reporting Summary [file 41467_2022_28719_MOESM2_ESM.pdf]

nature research

Corresponding author(s): Gene W. Yeo

Last updated by author(s): Jan 30, 2022

Reporting Summary

Nature Research wishes to improve the reproducibility of the work that we publish. This form provides structure for consistency and transparency in reporting. For further information on Nature Research policies, see our Editorial Policies and the Editorial Policy Checklist.

Statistics

For all statistical analyses, confirm that the following items are present in the figure legend, table legend, main text, or Methods section.

n/a	Confirmed	
☐	☒	The exact sample size (*n*) for each experimental group/condition, given as a discrete number and unit of measurement
☐	☒	A statement on whether measurements were taken from distinct samples or whether the same sample was measured repeatedly
☐	☒	The statistical test(s) used AND whether they are one- or two-sided<br>*Only common tests should be described solely by name; describe more complex techniques in the Methods section.*
☒	☐	A description of all covariates tested
☒	☐	A description of any assumptions or corrections, such as tests of normality and adjustment for multiple comparisons
☐	☒	A full description of the statistical parameters including central tendency (e.g. means) or other basic estimates (e.g. regression coefficient) AND variation (e.g. standard deviation) or associated estimates of uncertainty (e.g. confidence intervals)
☐	☒	For null hypothesis testing, the test statistic (e.g. *F*, *t*, *r*) with confidence intervals, effect sizes, degrees of freedom and *P* value noted<br>*Give P values as exact values whenever suitable.*
☒	☐	For Bayesian analysis, information on the choice of priors and Markov chain Monte Carlo settings
☒	☐	For hierarchical and complex designs, identification of the appropriate level for tests and full reporting of outcomes
☒	☐	Estimates of effect sizes (e.g. Cohen's *d*, Pearson's *r*), indicating how they were calculated

Our web collection on statistics for biologists contains articles on many of the points above.

Software and code

Policy information about availability of computer code

Data collection	Zeiss ZEN 2010 software was used to generate maximum intensity projection IF images taken by the Zeiss LSM 780.
Data analysis	eCLIP-Seq data were processed through Dr. Yeo's eCLIP pipeline version 0.4.0 (https://github.com/YeoLab). Custom scripts written in Python 3.7.7 and MATLAB 2019b were used to analyze data further. ViennaRNA-2.4.17 was used to analyze RNA secondary structures. Other software used for genomic analysis includes bowtie (1.2.2), bedtools (2.27.1), BWA (0.7.17), and the GUIDE-Seq pipeline (https://github.com/tsailabSJ/guideseq). Proteins were quantified using ImageJ Version 2.0.0-rc-69/1.52n (https://imagej.nih.gov/).

For manuscripts utilizing custom algorithms or software that are central to the research but not yet described in published literature, software must be made available to editors and reviewers. We strongly encourage code deposition in a community repository (e.g. GitHub). See the Nature Research guidelines for submitting code & software for further information.

Data

Policy information about availability of data

All manuscripts must include a data availability statement. This statement should provide the following information, where applicable:

- Accession codes, unique identifiers, or web links for publicly available datasets
- A list of figures that have associated raw data
- A description of any restrictions on data availability

The sequencing data generated in this study have been deposited in the NCBI GEO (Gene Expression Omnibus) database under accession code GSE167466. All uncropped EMSA and Western blot gel image files critical to the manuscript have been made available in the Supplementary Information.

Field-specific reporting

Please select the one below that is the best fit for your research. If you are not sure, read the appropriate sections before making your selection.

- [x] Life sciences
- [ ] Behavioural & social sciences
- [ ] Ecological, evolutionary & environmental sciences

For a reference copy of the document with all sections, see nature.com/documents/nr-reporting-summary-flat.pdf

Life sciences study design

All studies must disclose on these points even when the disclosure is negative.

Sample size	Sample sizes were determined according to the standards of the field. Typically two biological replicates are selected in CLIP experiments (Van Nostrand et al.,2016). In cases where statistical analysis was presented (i.e., RT-qPCR and Western blots), experiments were performed in three replicates. In addition, EMSAs were performed independently in triplicate with similar results, with a representative image shown.
Data exclusions	We did not exclude any data under consideration, except in instances when appropriate filters, as described in the text, were applied. These include excluding: (1) irreproducible eCLIP peaks in Fig. 1, (2) lowly expressed (< 1 Log2 RPKM in control) Cas9-mediated gene expression data in Supplementary Fig. 3, (3) irreproducible icSHAPE data in Supplementary Fig. 7, (4) lowly expressed (<1 TPM) genes in Supplementary Fig. 9, and (5) eCLIP peaks not coinciding with publicly available datasets for Fig. 3 and Supplementary Figs. 8 and 9.
Replication	Reported experimental results were replicated independently in triplicate consistently among replicates, with the exception of eCLIP results which were replicated independently in duplicate consistently between replicates.
Randomization	We did not use randomization of samples in this study. Randomization is generally not employed in this field. All experiments were well controlled with appropriate negative controls, as described in the text. Covariate control is not relevant to our study, because appropriate experimental negative controls were used to detect potential confounding and bias.
Blinding	Investigators were not blinded to the identities of samples in this study. Blindness is generally not employed in this field. All experiments were well controlled with appropriate negative controls, as described in the text. Blinding is not relevant to our study, because none of our collected data is clinical in nature and none of our authors had preconceived notions about experimental outcomes.

Reporting for specific materials, systems and methods

We require information from authors about some types of materials, experimental systems and methods used in many studies. Here, indicate whether each material, system or method listed is relevant to your study. If you are not sure if a list item applies to your research, read the appropriate section before selecting a response.

Materials & experimental systems

n/a	Involved in the study	
☐	☒	Antibodies
☐	☒	Eukaryotic cell lines
☒	☐	Palaeontology and archaeology
☒	☐	Animals and other organisms
☒	☐	Human research participants
☒	☐	Clinical data
☒	☐	Dual use research of concern

Methods

n/a	Involved in the study	
☒	☐	ChIP-seq
☒	☐	Flow cytometry
☒	☐	MRI-based neuroimaging

Antibodies

Antibodies used	The following antibodies were used: V5 Tag mouse monoclonal antibody (ThermoFisher Scientific #R960-25) at 1:3000 dilution, mouse monoclonal ANTI-FLAG M2 antibody (Sigma-Aldrich #F1804) at 1:3000 dilution, V5 Tag mouse monoclonal antibody (ThermoFisher Scientific #R960-25) at 1:3000 dilution, mouse monoclonal ANTI-FLAG M2 antibody (Sigma-Aldrich #F1804) at 1:1000 dilution, goat anti-mouse IgG AlexaFluor 488 Superclonal Recombinant Secondary antibody (ThermoFisher Scientific #A28175) at 1:2000 dilution, primary antibody Recombinant Anti-ATF3 antibody [EPR22610-19] (Abcam #ab254268) at 1:1000 dilution, secondary antibody IRDye 680RD Goat anti-Rabbit IgG Secondary Antibody (Li-Cor #926-68071) at 1:20,000 dilution, primary antibody Anti-alpha Tubulin antibody [DM1A] - Loading Control (Abcam #ab7291) at 1:5000 dilution, and secondary antibody IRDye 800CW Goat anti-Mouse IgG Secondary Antibody (Li-Cor #926-32210) at 1:20,000.
Validation	Primary antibodies are regularly validated through immunoprecipitation and Western blot of lysate from HEK 293T cells transfected with either V5/3xFLAG-tagged protein (positive control) or empty vector (negative control). Likewise, secondary antibodies for IF are validated on HEK 293T cells transfected with either V5/3xFLAG-tagged protein (positive control) or empty vector (negative control). ATF3 and alpha-tubulin antibodies were validated in HEK 293T cell lysate by weight/size according to a protein ladder.

Eukaryotic cell lines

Policy information about cell lines

Cell line source(s)	Lenti-X 293T (HEK 293T) Cell Line, purchased from Takara Bio.
Authentication	Cell lines have been authenticated by the vendors and have been re-authenticated by STR assay prior to cryobanking in the lab.
Mycoplasma contamination	Mycoplasma testing is routinely performed every 3 passages. All cells used in this study tested negative for mycoplasma contamination.
Commonly misidentified lines (See ICLAC register)	No commonly misidentified cell lines were used in the study.
