# Supplementary material for: Crosstalk between CRISPR-Cas9 and the human transcriptome
Source: Nat Commun. 2022 Mar 2;13:1125. doi: 10.1038/s41467-022-28719-5 (PMC8891275; doi:10.1038/s41467-022-28719-5)
Supplement: Supplementary file 3 — Description of Additional Supplementary Files [file 41467_2022_28719_MOESM3_ESM.pdf]

Title: Supplementary Data 1.
Description: List of all eCLIP peaks (n=478) for V5/FLAG dSpCas9 eCLIPs intersection in Fig. 1a, with chromosomes, peak interval starts, peak interval ends, and strands as aligned to human reference genome hg38.

Title: Supplementary Data 2.
Description: List of all eCLIP genes (n=381) for V5/FLAG dSpCas9 eCLIPs intersection in Fig. 1a, with total number of peaks per gene and number of peaks represented in each region per gene. Certain peaks span multiple regions in V5 and/or FLAG dSpCas9 eCLIPs, which explains why the number of peaks represented in each region per gene may not add up to the total number of peaks per gene.
